# Licochalcone A mitigates cisplatin-induced nephrotoxicity by inhibiting ferroptosis and mitochondrial dysfunction via the Nrf2/GPX4 axis

**DOI:** 10.3389/fphar.2026.1759824

**Published:** 2026-04-17

**Authors:** Mo Han, Feihu Zhang, Mengyuan Li, Qixiang Zhang, Kaidi Shen, Depeng Zhang, Huanke Xu, Fang Zhou, Xinyu Wang

**Affiliations:** 1 Key Laboratory of Drug Metabolism and Pharmacokinetics, State Key Laboratory of Natural Medicines, China Pharmaceutical University, Nanjing, China; 2 Department of Pharmacy and Institute of Clinical Pharmacology, General Hospital of Ningxia Medical University, Yinchuan, China

**Keywords:** cisplatin-induced nephrotoxicity, ferroptosis, LicoA, mitochondrion, Nrf2

## Abstract

**Background:**

Cisplatin-induced nephrotoxicity is a major dose-limiting complication of chemotherapy. However, effective protective strategies remain limited. Licochalcone A (LicoA), a flavonoid isolated from Glycyrrhiza inflata, exhibits multi-target bioactivity against oxidative stress, inflammation, and metabolic disorders.

**Methods:**

*In vivo* murine cisplatin-induced model and *in vitro* HK-2 cells were employed. HE/Masson staining, renal function, oxidative stress, ferroptosis markers (Fe^2+^, GSH, MDA, lipid peroxidation), and mitochondrial health were assessed. Mechanistic studies were conducted using metabolomic approaches, molecular docking, cellular thermal shift assay, Western blot, immunofluorescence, and Nrf2-knockdown assays.

**Results:**

LicoA significantly ameliorated cisplatin-induced renal dysfunction and attenuated the progression from acute kidney injury (AKI) to chronic kidney disease (CKD), as evidenced by reduced tubular injury and fibrosis. Metabolomic analysis revealed that LicoA restored energy metabolism and glutathione homeostasis. LicoA effectively alleviated cisplatin-induced ferroptosis by inhibiting Fe^2+^ accumulation, reducing lipid peroxidation, restoring cellular redox homeostasis, and consequently diminishing membrane blistering. Molecular docking and cellular thermal shift assay confirmed the binding affinity between LicoA and both Nrf2 and GPX4. Crucially, Nrf2 knockdown abolished the protective effects of LicoA, demonstrating the essential role of the Nrf2 pathway.

**Conclusion:**

LicoA alleviated cisplatin-induced renal injury by activating the Nrf2/GPX4 axis, thereby suppressing ferroptosis and mitigating mitochondrial damage. These findings highlight the therapeutic potential of LicoA in preventing cisplatin nephrotoxicity and impeding the AKI-to-CKD transition.

## 1 Introduction

Cisplatin combination therapy is a primary approach for solid malignancies (Tang et al., 2023; van der Heijden et al., 2023; Zhang et al., 2019), but its clinical application is limited by cumulative nephrotoxicity (Pabla and Dong, 2008). Cisplatin induces AKI within days in 30% of patients post-treatment, and multi-course regimens drive CKD progression (Dasari and Tchounwou, 2014; Latcha et al., 2016). Clinical studies indicate that cisplatin exposure has been linked to increased CKD incidence and progression in cancer survivors (Bhat et al., 2015; Duan et al., 2020). Despite decades of research, few FDA-approved protective strategies exist. Amifostine has been approved for mitigating nephrotoxicity associated with cisplatin-based chemotherapy (Li et al., 2024). However, its clinical use remains limited due to adverse effects such as hypotension, nausea, vomiting, and somnolence. As a result, current standard clinical management continues to rely primarily on hydration protocols and close monitoring of electrolyte balance (Tang et al., 2023). Thus, there is an urgent need for novel therapies to treat cisplatin-induced nephrotoxicity and its CKD transition.

Excessive oxidative stress is a pivotal contributor to cisplatin-induced nephrotoxicity, with the proximal tubule being the primary target (Fujishiro et al., 2021). In this segment, cisplatin triggers a multifaceted injury cascade involving DNA damage (Basu and Krishnamurthy, 2010; Wang and Lippard, 2005), oxidative stress, mitochondrial dysfunction, and diverse cell death pathways such as ferroptosis (Chiba et al., 2019). Ferroptosis, characterized by iron accumulation and phospholipid peroxidation (Dixon et al., 2012), contributes to the pathogenesis of AKI, involving tubular cell regeneration and interstitial fibrosis, and has become a promising therapeutic target for kidney injury (Hu et al., 2022). Previous studies have demonstrated that nuclear factor erythroid 2-related factor 2 (Nrf2) transcriptionally regulates virtually all ferroptosis-related genes, including, but not limited to glutathione peroxidase 4 (GPX4), as well as iron homeostasis mediators (Qu et al., 2025; Zhang et al., 2024). As a key selenoprotein, GPX4 eliminates phospholipid hydroperoxides and is crucial for suppressing ferroptosis. Nevertheless, the exact role of the Nrf2/GPX4 signaling pathway in nephrotoxicity after cisplatin exposure has not been fully elucidated.

**FIGURE 1:**
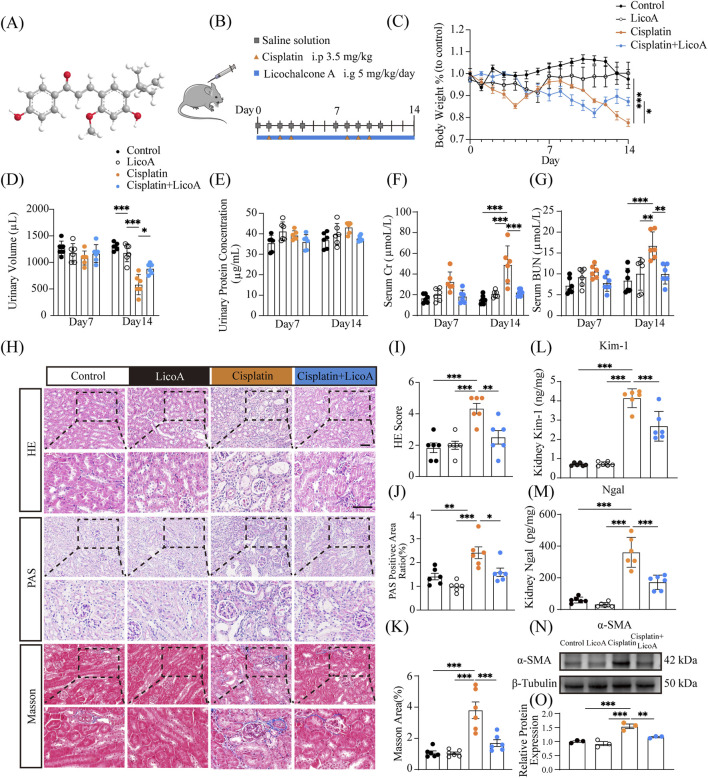
LicoA ameliorates cisplatin-induced nephrotoxicity *in vivo*. **(A)** The chemical structure of LicoA. **(B)** Experimental Flowchart. **(C)** Body weight. **(D)** Urinary volume. **(E)** Urinary protein level. **(F,G)** sCr and BUN. **(H)** Representative H&E, PAS, and Masson staining images of renal tissue (scale bar = 50 μm). **(I)** HE score, **(J)** The percentage of positive area of PAS stained in the spheres. **(K)** Quantification of renal relative fibrotic area. **(L,M)** The protein quantitation of Kim-1 and Ngal in mouse kidneys at Day 14. **(N,O)** The protein expression level of α-SMA at Day 14, n = 3. Results are expressed as means ± SEM, all n = 6 per group unless stated. *p < 0.05, **p < 0.01, ***p < 0.001.

The root of G. inflata Batal has long been regarded as an important source of licorice and is widely used in Asia and around the world. Licochalcone A (LicoA), a bioactive chalcone flavonoid from Glycyrrhiza inflata (Figure 1A), exhibits potent antioxidant activities by modulation of Nrf2 pathways and ferroptosis inhibition (Su et al., 2018; Li M.-T. et al., 2022; Lin et al., 2019; Lin et al., 2023). Preclinical studies demonstrate its protection against cisplatin-induced nephrotoxicity (Lee et al., 2008) without reducing antitumor activity, along with effectiveness in LPS-induced AKI(Hu and Liu, 2016). Notably, recent evidences reveal LicoA suppresses ferroptosis in cardiac ischemia-reperfusion injury and aflatoxin B1-induced immunotoxicity (Lin et al., 2023). However, its efficacy in attenuating AKI-to-CKD transition and the potential involvement of ferroptosis suppression in its renoprotective mechanism remain unexplored.

Herein, we aimed to investigate the therapeutic effect of LicoA on cisplatin-induced kidney injury, as well as its impact on ferroptosis through the Nrf2/GPX4 axis. This study seeks to elucidate the mechanistic basis for LicoA-mediated renoprotection and provides novel insights into the management of cisplatin-induced nephrotoxicity.

## 2 Materials and methods

### 2.1 Chemicals and materials

Cisplatin, characterized by a platinum content exceeding 98%, and LicoA were procured from Shanghai Yuanye Biotechnology Co. (Shanghai). Ferrostatin-1 were procured from MCE (MCE, USA). DMEM and trypsin were sourced from Gibco (Grand Island, USA), while fetal bovine serum (FBS) was acquired from Genetimes Biotechnology Co. (Shanghai). PCR reaction reagents were purchased from Vazyme Biotech Co., Ltd. (Nanjing), and primers were purchased from Songon Biotech (Shanghai). All other reagents were commercially obtained.

### 2.2 Animal experiments

We used male C57BL/6J mice, aged 8 weeks and weighing 20–22 g (Vital River Laboratory Animal Technology, Zhejiang). The rodents were housed under a 12-h light/dark cycle at a constant temperature of 25 °C, with access to food and water. After a week of acclimatization, 24 mice were randomly allocated into four groups of 6 animals each: (1) Control (received saline intraperitoneally daily); (2) Cisplatin (administered at 3.5 mg/kg intraperitoneally on days 1–3 of each 7-day cycle); (3) LicoA (given at 5.0 mg/kg intragastrically daily); (4) Cisplatin + LicoA (received LicoA at 5.0 mg/kg intragastrically daily plus Cisplatin, as per group 2). A total of two cycles were carried out, with subcutaneous saline hydration (1 mL/day) initiated 1 day prior to Cisplatin administration and continued for five consecutive days. The mice were euthanized through spinal dislocation under isoflurane anesthesia, with kidneys fixed in 4% paraformaldehyde/3% glutaraldehyde or frozen at −80 °C, and serum samples were collected.

### 2.3 Biochemical assays

Serum creatinine (sCr), blood urea nitrogen (BUN), intracellular/renal GSH, MDA, and Fe^2+^ levels were measured using colorimetric kits (Elabscience, Wuhan, Hubei, China).

### 2.4 Elisa assays

Protein levels of Kim-1 and Ngal in serum and kidney tissues were quantified using commercially available ELISA kits (Elabscience, Wuhan, Hubei, China). Sample pretreatment was performed according to the manufacturer’s manual.

### 2.5 Histopathology assessment

Renal tissue sections were processed for H&E and PAS staining by the Pathology and PDX Efficacy Evaluation Center at China Pharmaceutical University. We focused on the renal cortex of 6 samples per group for all pathological evaluations, including fibrosis and tubular injury, as it is the region most affected by cisplatin-induced nephrotoxicity. For each sample, we examined a minimum of 3 random fields in each section to ensure comprehensive analysis. This allowed for reliable quantification of tissue damage. The analysis was performed using ImageJ (2.14.0) software to quantify the areas of injury and fibrosis.

The degree of renal injury was assessed using the Hematoxylin and Eosin (HE) and Periodic Acid-Schiff (PAS) staining. For HE, the scoring system evaluated tubular injury, including loss of brush border, tubular dilation, and cast formation. The degree of injury was graded on a scale from 1 to 5 as follows: 1, no injury; 2, mild injury (A small number of tubules exhibited loss of the brush border or mild tubular dilation, with no cast formation observed.); 3, moderate injury (Many tubules exhibited loss of the brush border and marked tubular dilation, with occasional cast formation); 4, severe injury (Widespread loss of the brush border and tubular dilation in tubules, with cast formation commonly observed.); and 5, very severe injury (Severely disrupted tubular structure, with widespread loss of the brush border, tubular dilation, and extensive cast formation, accompanied by shedding of renal tubular epithelial cells). For PAS staining, semi-quantitative analysis was performed by calculating the percentage of the PAS-positive area relative to the total glomerular area. We outlined the contour of a single glomerulus along the inner or outer edge of Bowman’s capsule using the freehand selection tool. Subsequently, PAS-positive areas were segmented using the HSB thresholding method, with the red overlay adjusted to precisely select these regions within the glomerulus for further analysis. A minimum of 3 images were captured per sample, and 3–5 glomeruli were measured repeatedly per image. The mean value was calculated for each sample, with 6 samples included per group. Fibrosis in kidney tissues was assessed using Masson’s trichrome staining. Fibrosis quantification was performed using the HSB thresholding method, based on the percentage of the renal area stained positively for collagen fibers.

### 2.6 Quantitative Real-Time PCR analysis

Total RNA was extracted with TRIzol reagent, reverse-transcribed to cDNA, and amplified using SYBR Green chemistry on a CFX96 Real-Time PCR System (Bio-Rad, USA). Target gene expression was normalized to β-actin, with primer sequences detailed in Table 1.

**TABLE 1:** Primer sets used in PCR analysis.

*Gene symbol*	*Primers*
*β-actin*	*F: ACG​GCC​AGG​TCA​TCA​CTA​TTG*
	*R: AGG​GGC​CGG​ACT​CAT​CGT​A*
*Kim-1*	*F: AGC​AGT​CGG​TAC​AAC​TTA​AAG​G*
	*R: ACT​CGA​CAA​CAA​TAC​AGA​CCA​C*
*Ngal*	*F: GCA​GGT​GGT​ACG​TTG​TGG​G*
	*R: CTC​TTG​TAG​CTC​ATA​GAT​GGT​GC*
*α-SMA*	*F: CCC​AGA​CAT​CAG​GGA​GTA​ATG​G*
	*R: TCT​ATC​GGA​TAC​TTC​AGC​GTC​A*

### 2.7 Western blot

The cellular and tissue samples were homogenized on ice using RIPA Lysis buffer supplemented with protease inhibitors (Beyotime). After a 10-min centrifugation at 14,000 g and 4 °C, the supernatant was carefully collected. Protein concentrations were then determined using the BCA Protein Assay Kit (Beyotime) according to the manufacturer’s protocol.

Samples (50 μg kidney/30 μg cell protein) were separated via SDS-PAGE. After that, samples were transferred to polyvinylidene fluoride membranes (Millipore, Bedford, USA). After blocking with 5% BSA for 1 h, the membranes were incubated with anti-α-SMA (1:1,000 dilution, Abcam, USA) anti-Nrf2 (1:1,000 dilution, Proteintech Group, Rosemont, IL, USA), anti-HO-1 (1:1,000 dilution, Proteintech Group, Rosemont, IL, USA), anti-GPX4 (1:1,000 dilution, Abmart, Shanghai, China), anti-xCT (1:1,000 dilution, Shanghai, China), anti-β-tubulin antibodies (1:1,000 dilution, Cell Signaling Technology, Danvers, MA, USA) or anti-Lamin-B1 antibodies (1:10,000 dilution, Proteintech Group, Rosemont, IL, USA) at 4 °C overnight, followed by horseradish peroxidase-linked anti-mouse (1:1,000 dilution, Cell Signaling Technology, Danvers, MA, USA) or anti-rabbit secondary antibodies (1:2,000 dilution, Cell Signaling Technology, Danvers, MA, USA) at 37 °C for 1 h. The signals were detected with an enhanced chemiluminescence kit (Bio-Rad, Bio-Rad Laboratories, USA) and the signals were visualized with a ChemiDoc XRS+β system (Bio-Rad, Bio-Rad Laboratories, USA). Quantification was calculated by ImageJ 2.14.0 software and normalized to β-tubulin or Lamin-B1.

### 2.8 Immunofluorescence (IF) assay

HK-2 cells were plated on slides in 24-well plates. After drug treatment, the cells were treated using the protocol we have described (Chen B. et al., 2024). Following fixation in 4% paraformaldehyde (PFA) for 2 h at room temperature, kidneys were processed for frozen sectioning. For cryopreservation, specimens were immersed in 30% sucrose in phosphate-buffered saline (PBS) until they became submerged, subsequently embedded in Tissue-Tek O.C.T. compound (Sakura, USA), and the tissues were sectioned at 10 μm on cryostat (Leica German). The slides were incubated with primary antibodies (anti-Nrf2 [1:200 dilution], anti-HO-1 [1:200 dilution]). Following with the secondary antibody, CoraLite488-conjugated Goat Anti-Mouse/Rabbit IgG (1:1,000 dilution, Invitrogen, U.S.A.). All images were captured using the FV3000 confocal microscopy (Olympus, Japan).

### 2.9 Immunohistochemistry (IHC) assay

Kidney tissues were paraffin-embedded, sectioned, and deparaffinized in xylene, rehydrated through graded ethanol (100%–70%), followed by placement in PBS. Kidney sections were provided by Servicebio Technology. Sections were incubated with the primary antibodies xCT (1:200 dilution, Abmart, Shanghai, China) and GPX4 (1:200 dilution, Abmart, Shanghai, China). Images were acquired under a light microscope.

### 2.10 Transmission electron microscopy (TEM)

Fresh kidney tissue was cut into small pieces and incubated in fixing solution at 4 °C for 4 h in preparation for transmission electron microscopy, followed by secondary fixation at room temperature using 0.1 mol/L of PBS containing 1% osmium tetroxide for 2 h. After these treatments, the samples were dehydrated using an ethanol gradient series. Subsequently, the sections were embedded and polymerized at 60 °C for 48 h. Ultrathin sections (60 nm) were prepared using an ultramicrotome and imaged via TEM (Hitachi, Japan). We analyzed three kidney samples per group. From each sample, three randomly selected fields were assessed to ensure sufficient representation of mitochondrial morphology. These fields were chosen from the renal cortex. Ultrastructural analysis via transmission electron microscopy followed established protocols (Wang et al., 2022).

### 2.11 Cell culture and administration

HK-2 cells, procured from Zhong Qiao Xin Zhou Biotechnology Co. (Shanghai), were nurtured in DMEM medium supplemented with 10% fetal bovine serum. They were kept were maintained at 37 °C within a climate-controlled environment with a 5% CO2 atmosphere. Once they reached the peak of their growth, we split them into four distinct groups. The Control group got the standard treatment—a simple aqueous saline solution. The Cisplatin group was treated with 20 μM of the drug. The LicoA + Cisplatin group received both 20 μM of Cisplatin and 4 μM of LicoA. Lastly, the LicoA group was dosed with just 4 μM of LicoA.

### 2.12 Bodipy (581/591)-C11 and MitoTracker® deep red FM staining

Bodipy (581/591)-C11 (Invitrogen, U.S.A.) and MitoTracker® Deep Red FM were used to detect lipid peroxidation and mitochondrial localization, respectively. Cells were incubated with a mixture of 5 mM Bodipy (581/591)- C11, 0.2 mmol/L Mitotracker (Invitrogen), and 0.5 mg/mL Hoechst (Beyotime, Shanghai, China). In serum-free culture medium for 20 min at 37 °C, then imaged using FV3000 confocal microscopy (Olympus, Japan) for observation. Mitochondrial skeleton analysis was assessed via Mitochondria Analyzer using ImageJ 2.14.0 software (National Institutes of Health, U.S.A.).

### 2.13 ImageJ-based mitochondrial skeleton analysis

Mitochondrial morphology was assessed by analyzing the mitochondrial network skeleton using ImageJ 2.14.0 software. Confocal fluorescence images of mitochondria-stained cells were acquired. All image processing steps were performed using a custom macro to ensure batch consistency, with the detailed workflow as follows. First, raw images were converted to 8-bit grayscale. Background was subtracted using a rolling ball radius of 50 pixels. A median filter (radius 1 pixel) was applied to reduce noise while preserving mitochondrial structures. Images were then set the same threshold method to generate binary masks. The binary images were subjected to the “Skeletonize” function (2D/3D) to reduce mitochondrial tubules to single-pixel-wide representations. Subsequently, the “Analyze Skeleton” plugin (version 2.1.0) was employed to quantify the skeletonized networks. Six random fields of view were selected for each group, with 3–5 cells analyzed per field, resulting in a total of 16–25 cells for Mitochondrial Skeleton Analysis.

### 2.14 ROS staining

Cell culture medium was replaced with the appropriate volume of diluted 10 mM. DCFH-DA (Beyotime, Shanghai, China), followed by co-incubation with Hochest (Beyotime, Shanghai, China) at 37 °C for 20 min. After being washed three times with serum-free medium, images were acquired using the BioTek LionheartFX imaging system (Biotek).

### 2.15 Mitochondrial membrane potential detection

Following the established protocol, cells were incubated in a fresh JC-1 working solution (Beyotime, Shanghai, China) at 37 °C for 20 min. The cells were then washed with PBS, and the fluorescence ratio was subsequently quantified using ImageJ software (version 2.14.0, NIH, USA).

### 2.16 Metabolomics studies

Metabolomic analysis was carried out using LC/Q-TOF MS. For sample pretreatment, L-Glutamine-^13^C_5_ (MCE, USA) was used as the internal standard (IS) at a concentration of 1.5 μg mL^-1^ in all extraction solvents. 5 × 10^6^ HK-2 cells were homogenized in 500 μL of 80% methanol containing the IS (2.0 μg mL^-1^). After that, all samples were shaken for 10 min and then left to stand at 4 °C for 1 h to precipitate proteins. The supernatant was collected after centrifugation at 180,00 × g at 4 °C for 10 min. The solvent was evaporated under reduced pressure using a vacuum centrifuge and a rotary evaporator. The dried residue was reconstituted in 100 μL of ultrapure water. The reconstituted samples were centrifuged at 18,000 rpm for 10 min at 4 °C, and the supernatant was transferred to vials for LC–MS analysis.

Agilent HPLC system coupled with a Triple TOF 5600 (AB SCIEX) was applied for chromatographic separation. Waters Amide XBridge HPLC column (3.5 μm; 4.6 mm × 100 mm, Waters) was used. Mobile phase A consisted of 95% of 5 mM ammonium acetate (pH adjusted to 9.0 with 10% ammonia) mixed with 5% of acetonitrile; mobile phase B was 100% acetonitrile. Gradient elution was carried out as follows: 0–3 min, 85% B; 3–6 min, 85%–30% B; 6–15 min, 30%–2% B; 15–18 min, 2% B; 18–19 min, 2%–85% B; 19–26 min, 85% B. The injection volume was 10 μL, the column temperature was maintained at 35 °C, and the flow rate was 0.4 mL min^-1^. The mass spectrometer was operated in negative ion mode. Full-scan MS spectra were acquired over an m/z range of 50–1,000, and MS/MS spectra were recorded over m/z 50–900. Raw data were processed using Analyst TF 1.7 and MultiQuant 3.0 software (AB SCIEX). The resulting multivariate data were subjected to partial least squares discriminant analysis (PLS-DA), and differential metabolites were further explored through pathway analysis using MetaboAnalyst (www.metabonalyst.ca, accessed 6 June 2021).

### 2.17 Cell transfection

Cells were transfected with either Nrf2-siRNA or a negative control siRNA (NC-siRNA) (GenePharm, Shanghai, China) using Lipofectamine™ RNAiMAX reagent (Invitrogen, Carlsbad, CA, USA), following the previously detailed methodology (Chen B. et al., 2024).

### 2.18 Molecular docking

Molecular docking was performed to predict the binding modes of Licochalcone A (LicoA) with Nrf2, GPX4, xCT, and HO-1. The two-dimensional (2D) structure of LicoA was retrieved from the PubChem database (https://pubchem.ncbi.nlm.nih.gov/, accessed 20 October 2021) and subsequently converted into three-dimensional (3D) format. The crystal structures of Nrf2 (PDB ID: 7K2F), GPX4 (PDB ID: 7U4N), xCT (PDB ID: 7P9U), and HO-1 (PDB ID: 1N3U) were downloaded from the RCSB Protein Data Bank (https://www.rcsb.org/, accessed 21 October 2021).

Proteins were prepared using AutoDock Tools (version 1.5.6, The Scripps Research Institute), removing water molecules and non-standard residues, adding polar hydrogens, and assigning Gasteiger charges. Ligand flexibility was accounted for, and torsion and rotatable bonds were assigned. Grid boxes (100 × 100 × 100 points, 0.375 Å spacing) were defined around binding sites, and docking simulations were performed with AutoDock4 (The Scripps Research Institute) using the Lamarckian Genetic Algorithm (LGA). For each complex, 100 independent runs were performed with parameters: population size 150, max energy evaluations 2.5 million, max generations 27,000, mutation rate 0.02, and crossover rate 0.8. Poses were clustered by RMSD (2.0 Å) and ranked by estimated free energy of binding (ΔG, kcal/mol). PyMOL (version 2.5, Schrödinger, LLC) was used for visualization, with hydrogen bonds, hydrophobic, and π-π stacking interactions identified.

### 2.19 Cellular thermal shift assay

HK-2 cells were seeded into 10-cm dishes at a density of 3 × 10^6^ cells per dish 24 h prior to treatment. LicoA was dissolved in dimethyl sulfoxide (DMSO) to prepare a 10 mM stock solution and stored at −20 °C. For experiments, a working concentration of 4 μM was freshly prepared by diluting the stock solution in complete culture medium, yielding a final DMSO concentration of ≤0.1%. Vehicle control cells received an equivalent volume of DMSO in medium. Cells were incubated with LicoA or vehicle for 1 h at 37 °C.

After treatment, cells were washed twice with ice-cold PBS supplemented with protease inhibitor cocktail (Beyotime), then harvested by gentle scraping. Cell suspensions were transferred to pre-chilled 1.5-mL centrifuge tubes and pelleted by centrifugation at 400 × g for 5 min at 4 °C. Pellets were resuspended in 500 μL ice-cold PBS containing protease inhibitors, and 50 μL aliquots were distributed into 0.2-mL thin-wall PCR tubes. Thermal profiling was performed using a Veriti Thermal Cycler (Bio-Rad, Bio-Rad Laboratories, USA) across an 8-point temperature gradient (37.0, 41.0, 45.0, 49.0, 53.0, 57.0, 61.0, and 65.0 °C). Each sample was heated for 3 min, followed by a 3-min incubation at room temperature to permit protein refolding. Immediately after thermal challenge, 50 μL of ice-cold PBS containing 0.4% NP-40 (Beyotime) and protease inhibitors was added to each tube (final NP-40 concentration: 0.2%). Cells were lysed by gentle pipetting and incubated on a rotating shaker at 4 °C for 15 min. Lysates were centrifuged at 20,000 × *g* for 20 min at 4 °C, and the supernatants—containing soluble proteins—were carefully collected into new pre-chilled tubes for subsequent Western blot analysis.

### 2.20 Statistics analysis

Data are expressed as mean ± standard error of the mean (SEM). Statistical significance was assessed using unpaired Student’s t-tests (assuming equal variance), one-way or two-way ANOVA, followed by Sidak’s *post hoc* test for multiple comparisons. All analyses were conducted in GraphPad Prism (version 10), with a p-value < 0.05 considered statistically significant. All experiments were performed in triplicate for reproducibility.

## 3 Results

### 3.1 LicoA ameliorates cisplatin-induced nephrotoxicity *in vivo*

In a preliminary experiment, we investigated the effects of three distinct doses of LicoA (2.5 mg/kg, 5 mg/kg, and 10 mg/kg) on cisplatin-induced renal dysfunction. The results demonstrated that the group administered with 5 mg/kg of LicoA exhibited the least reduction in body weight compared to the other treatment groups. Moreover, serum creatinine levels were significantly lower in this group than in the cisplatin-treated control group (Supplementary Figures S1A–C). Therefore, the 5 mg/kg dose was chosen for further experimental validation. LicoA (5 mg/kg) was administered 1 day before cisplatin and maintained for 14 days, with cisplatin administered for 3 consecutive days followed by 4 days off for 2 cycles (Figure 1B). Cisplatin induced appetite loss and weight reduction from day 2, which was alleviated by LicoA. After two cycles, the Cisplatin + LicoA group exhibited about 10% higher body weight than the cisplatin group (Figure 1C). Urine volume decreased markedly in cycle 2 but was restored by LicoA (Figure 1D), while urinary protein remained unchanged (Figure 1E), indicating predominant tubular rather than glomerular injury. Critically, LicoA preserved renal function, as shown by BUN and sCr (Figures 1F,G; Supplementary Figures S1B,C). It also suppressed the mRNA (Supplementary Figures S1D,E) and protein (Figures 1L,M) expression of renal injury biomarkers kidney injury molecule 1 (Kim-1) and neutrophil gelatinase-associated lipocalin (Ngal). Besides, serum concentration of Kim-1 and Ngal was tested using elisa assay (Supplementary Figures S1G,H). Histologically, LicoA mitigated cisplatin’s nephrotoxic effects, including loss of brush border, cytoplasmic vacuoles, tubular dilation, and cast formation, while reducing collagen deposition in tubular basement membranes by approximately 50% (Figures 1H–K). Concomitantly, LicoA suppressed cisplatin-induced α-SMA expression (Figures 1N,O; Supplementary Figure S1F). The HE staining of tissues from the heart, liver, spleen, lung, and brain of 5 mg/kg LicoA dose group did not reveal any significant abnormalities or signs of toxicity (Supplementary Figure S1I). These results suggest that LicoA may ameliorate cisplatin-driven AKI to CKD progression. Collectively, these findings indicate that LicoA reduces cisplatin- induced renal impairment and tissue injury.

### 3.2 LicoA alleviates cisplatin-induced metabolic dysfunction and restores glutathione levels

**FIGURE 2:**
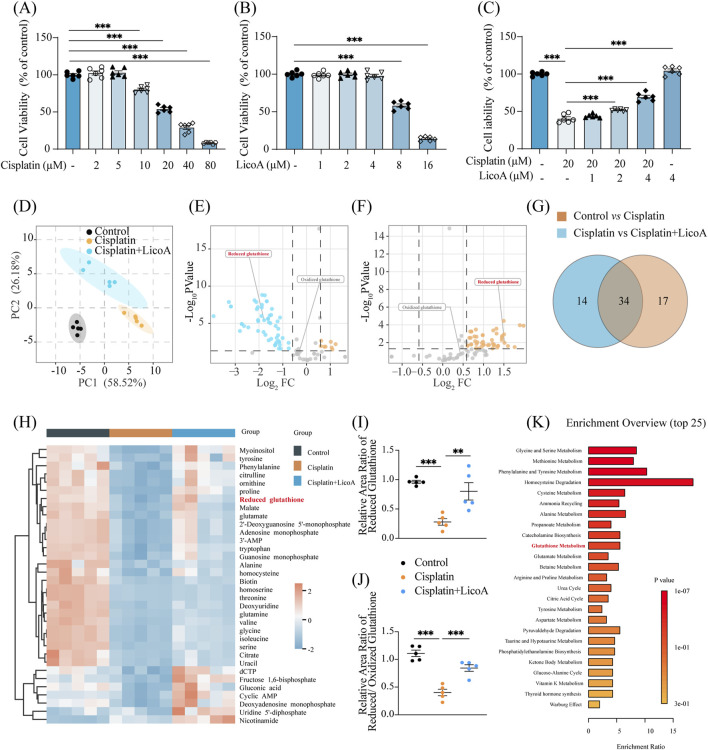
LicoA markedly improves cisplatin-related metabolic dysfunction and restores glutathione levels. **(A–C)** The viability of HK2 cells, cisplatin and/or LicoA were administered for 24 h, n = 6. **(D)** Partial Least Squares Discrimination Analysis (PLS-DA) plot. **(E,F)** Volcano maps analysis from the three assigned groups. **(G)** Venn diagram analysis. **(H)** Heat maps of differential compounds. **(I,J)** The levels of reduced glutathione, oxidized glutathione, and their ratio. **(K)** KEGG pathway analysis. Results are expressed as means ± SEM, *n* = 5. *p < 0.05, **p < 0.01, ***p < 0.001.

In this study, 20 μM cisplatin was selected to establish nephrotoxicity in HK-2 cells based on its significant inhibition of proliferation (Figure 2A). LicoA at 4 μM provided optimal protection against cisplatin-induced cytotoxicity, justifying its use in subsequent experiments (Figures 2B,C). From the perspective of cell metabolomics, we explored the potential mechanism of LicoA’s protective effect. PLS-DA analysis revealed distinct metabolic profiles between groups (Figure 2D). According to VIP >1 and p < 0.05 conditions, the qualified differential compounds were screened out. As illustrated in Figures 2E,F, LicoA co-treatment reversed cisplatin-induced depletion of reduced glutathione (GSH) and downregulation of TCA cycle intermediates, such as citrate, malate, and succinate. Among 48 cisplatin-altered metabolites, LicoA restored 34 to baseline levels (Figure 2G), including increased GSH and decreased oxidized glutathione (GSSG) (Figures 2H–J). Pathway analysis identified glutathione metabolism as a significantly correlated pathway (impact > 0.1, p < 0.001; Figure 2K).

### 3.3 LicoA protects HK-2 cells against cisplatin-triggered lipid peroxidation and ferroptosis

**FIGURE 3:**
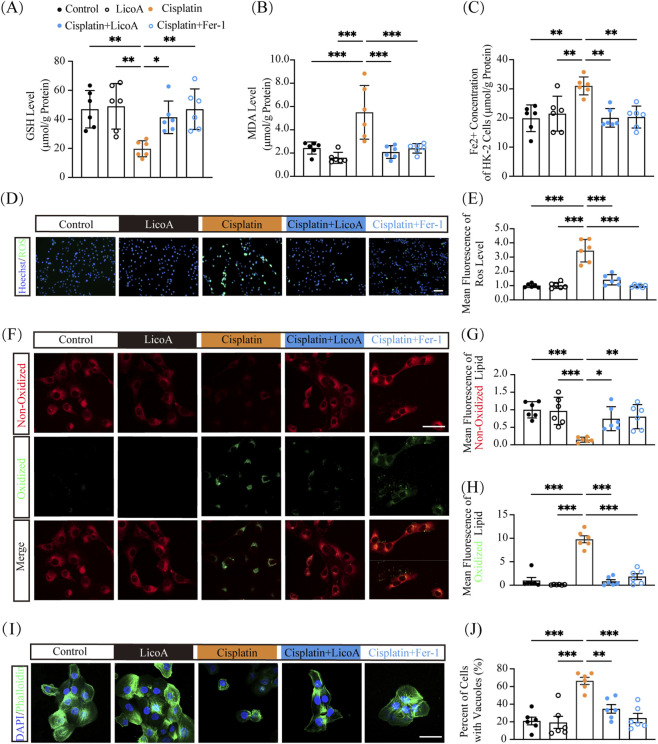
LicoA protects HK-2 cells against cisplatin-induced lipid peroxidation and ferroptosis. **(A)** GSH levels. **(B)** MDA levels. **(C)** Fe^2+^ levels. **(D,E)** ROS levels. **(F–H)** Lipid peroxidation levels. **(I,J)** Co-stained with phalloidin and DAPI. White arrows indicate the bulges of the cell membrane. The number of cells with blistered membranes was calculated. Scale bar = 50 μm. Results are expressed as means ± SEM, all n = 6 per group unless stated. *p < 0.05, **p < 0.01, ***p < 0.001.

Ferroptosis, an iron-dependent cell death driven by lipid peroxidation, was evaluated through key biomarkers including Fe^2+^ accumulation, GSH depletion, MDA elevation, ROS generation, and membrane integrity. Cisplatin exposure triggered profound ferroptotic responses in HK-2 cells, initiating with an increase in intracellular Fe^2+^ accumulation, which was effectively reversed by 4 μM LicoA co-treatment (Figure 3A). Concurrently, cisplatin induced severe glutathione depletion (Figure 3B), MDA elevation (Figure 3C), and ROS overproduction (Figures 3D,E). LicoA significantly mitigated all three perturbations, restoring cellular redox homeostasis. Furthermore, LicoA inhibited cisplatin-stimulated lipid peroxidation (Figures 3F–H). Ultrastructural analysis revealed that cisplatin-treated cells exhibited membrane blebbing without overt rupture (Figures 3I,J), a phenotype that was reversed by LicoA co-administration. Moreover, ferrostatin-1 (Fer-1, 5 μM), a well-established, ferroptosis-specific inhibitor, was included as a positive pharmacological control. Notably, Fer-1 significantly reversed cisplatin-induced ferroptotic alterations—including intracellular Fe^2+^ accumulation, GSH depletion, MDA and ROS elevation, BODIPY-C11–detected lipid peroxidation, and loss of plasma membrane integrity. These findings constitute a canonical pharmacological rescue phenotype, confirming ferroptosis inhibition and thereby strengthening the conclusion that LicoA exerts cytoprotection primarily through suppression of ferroptosis.

### 3.4 The protective effect of LicoA from cisplatin-induced ferroptosis is associated with the Nrf2/GPX4 axis in HK-2 cells

**FIGURE 4:**
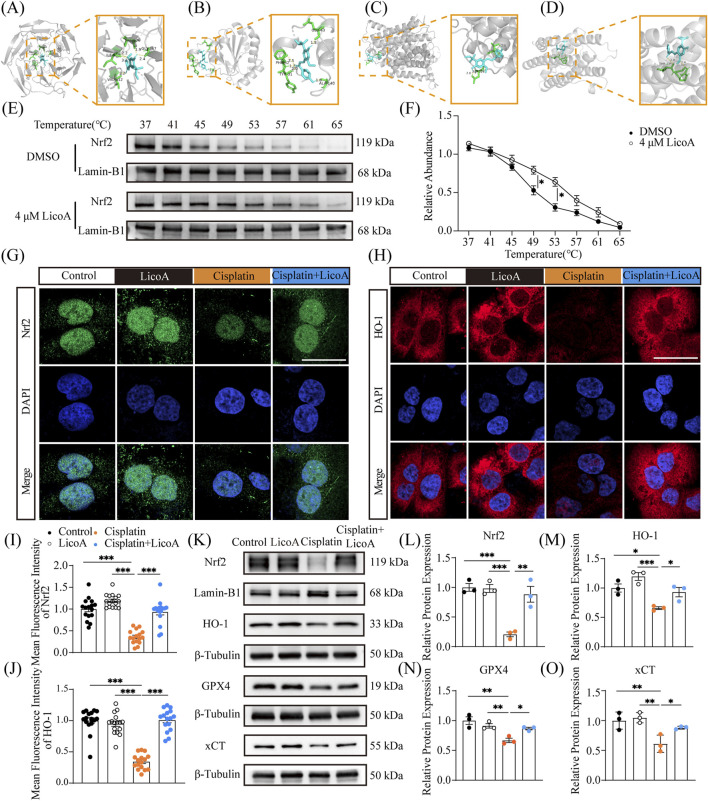
The protective effect of LicoA against cisplatin-induced ferroptosis is related to the Nrf2/GPX4 axis in HK-2 cells. **(A–D)** Molecular docking results of LicoA with Nrf2, GPX4, xCT, and HO-1. **(E,F)** CETSA protein expression and melting curve of Nrf2 in the presence of LicoA. **(G,H)** IF staining. Scale bar = 20 μm. **(I,J)** The mean fluorescence intensity, *n* = 16–25. **(K–O)** The protein expression of Nrf2, HO-1, xCT, and GPX4, *n* = 3. Results are expressed as means ± SEM. *p < 0.05, **p < 0.01, ***p < 0.001.

The Nrf2/HO-1 antioxidant axis is a central regulator of ferroptosis; its activation may protect against ferroptosis by suppressing oxidative stress and maintaining mitochondrial function. To explore LicoA’s protective mechanism, we evaluated its effect on the Nrf2/HO-1 axis and key ferroptosis regulators (xCT/SLC7A11 and GPX4). Molecular docking showed the strongest binding of LicoA to Nrf2 (Score = 9.20), forming hydrogen bonds with Nrf2 residues VAL-606 (1.9 Å), VAL-612 (2.2 Å), ILE-550 (2.3/3.2 Å), GLY-367 (2.4 Å) (Figure Fig4A). Conversely, LicoA exhibited weaker interaction with GPX4 (Score = 7.57), xCT (Score = 6.61) and HO-1 (Score = 4.77) (Figures 4B–D). To investigate whether LicoA directly binds to and stabilizes Nrf2, we performed CETSA. Cells were incubated with LicoA (or vehicle control) and subsequently subjected to heating across a temperature gradient (37.0 °C–65.0 °C). The results showed that (Figures 4E,F), with increasing temperature, the soluble fraction of Nrf2 gradually decreased in both groups, exhibiting typical thermal denaturation curves. However, compared to the control group, the LicoA-treated group significantly delayed the denaturation and precipitation of the Nrf2 protein within a specific temperature range (45.0 °C–65.0 °C). This was manifested as a pronounced rightward shift in the melting temperature of the protein, indicating that the binding of LicoA enhances the thermal stability of Nrf2. Immunofluorescence revealed that cisplatin inhibited Nrf2’s nuclear translocation (Figures 4G,I) and HO-1 activation, impairing the Nrf2/HO-1 axis, whereas LicoA restored the effects (Figures 4H,J). Additionally, LicoA significantly reversed the cisplatin-induced suppression of Nrf2, HO-1, GPX4, and xCT expression (Figures 4K–O). The direct cause of ferroptosis is the massive accumulation of lipid peroxides in the cell membrane, leading to membrane structure disruption. GPX4 is currently the only known enzyme that can effectively eliminate lipid peroxides from the membrane and is widely recognized as the core negative regulator of ferroptosis whose functions depends largely on the intracellular level of GSH (Jiang et al., 2021). We observed that LicoA upregulates GPX4 expression and restores or maintains GSH levels (Figure 3B), indicating that it likely enhances the function of this GSH- GPX4 defense system to resist ferroptosis. Collectively, these results indicate that LicoA protects against cisplatin-induced ferroptosis by activating the Nrf2/GPX4 antioxidant axis.

### 3.5 LicoA prevents the mitochondrial damage of HK-2 cells caused by cisplatin

**FIGURE 5:**
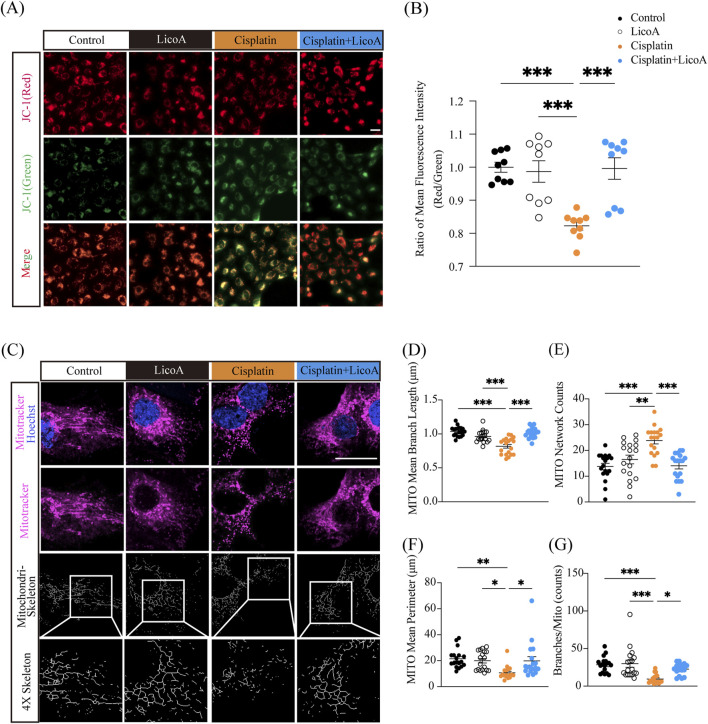
LicoA attenuates the mitochondrial damage of HK-2 cells caused by cisplatin. **(A,B)** MMP levels. Scale bar = 20 μm, *n* = 6. **(C)** Mitochondrial morphology. Scale bar = 20 μm. **(D–G)** Mitochondrial mean perimeter, networks, mean branch length, and individual branches. *n* = 16–25. Results are expressed as means ± SEM. *p < 0.05, **p < 0.01, ***p < 0.001.

We assessed mitochondrial membrane potential (MMP) and quantified mitochondrial network architecture using MitoTracker-based morphometric analysis to determine whether LicoA’s protection against cisplatin-induced ferroptosis extends to mitochondrial structure and function. As shown in Figures 5A,B, cisplatin exposure induced a significant loss of MMP—indicative of mitochondrial depolarization and early bioenergetic impairment. Notably, co-treatment with LicoA significantly attenuated this decline, suggesting partial preservation of mitochondrial functional integrity.

Mitochondrial network morphology was further evaluated. In control HK-2 cells, mitochondria formed an interconnected, reticular network characterized by elongated tubules and continuous branching. In contrast, cisplatin treatment triggered pronounced network fragmentation. Quantitative MitoTracker-based analysis revealed a significant increase in mitochondrial number and branch count, coupled with a marked decrease in mean branch length (Figures 5C–G), collectively reflecting reduced network connectivity and increased mitochondrial discontinuity. This remodeling is consistent with a shift in the fission–fusion balance toward excessive fission and/or compromised fusion dynamics under cisplatin-induced stress. Critically, LicoA co-treatment largely reversed these structural alterations: it normalized mitochondrial number and branch count, and restored mean branch length toward control levels—thereby rescuing network interconnectivity and promoting a more elongated, tubular architecture (Figures 5C–G). Together, these findings demonstrate that LicoA not only mitigates ferroptotic damage but also preserves mitochondrial structural homeostasis and functional polarization in cisplatin-induced HK-2 cells.

### 3.6 The inhibitory effect of LicoA on ferroptosis in HK-2 cells is dependent on the expression level of the Nrf2 gene

**FIGURE 6:**
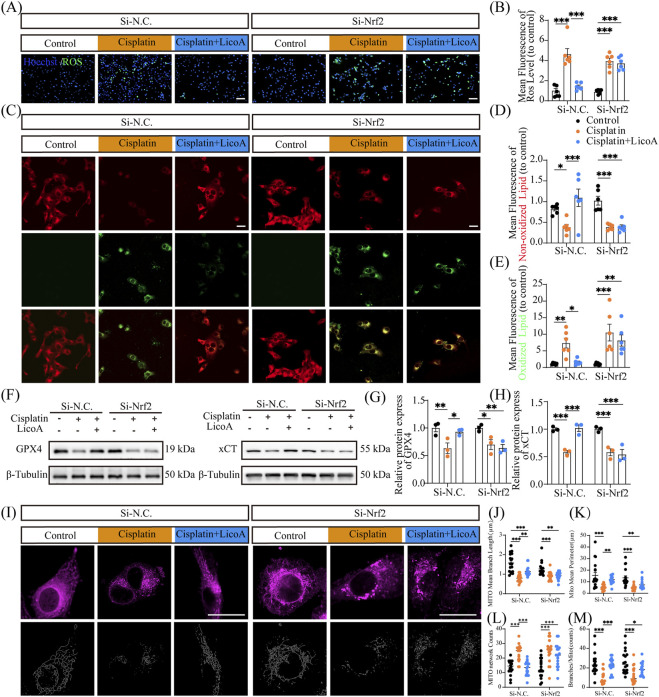
LicoA suppresses ferroptosis in HK-2 cells in an Nrf2-dependent manner. **(A,B)** ROS fluorescence levels. Scale bar = 50 μm, n = 6. **(C–E)** Lipid peroxidation levels. Scale bar = 20 μm, n = 6. **(F–H)** The protein expression of GPX4 and xCT in HK-2 cells, n = 3. **(I)** Mitochondrial morphology, Scale bar = 20 μm. **(J–M)** Mitochondrial mean perimeter, networks, mean branch length, and individual branches. *n* = 16–25, scale bar = 20 μm. Results are expressed as means ± SEM, *p < 0.05, **p < 0.01, ***p < 0.001.

To investigate the role of Nrf2 in LicoA-induced ferroptosis inhibition, Nrf2 protein expression in HK-2 cells was knocked down using Nrf2 siRNA (Supplementary Figures S2A,B). In Nrf2-knockdown cells, the inhibitory effects of LicoA on cellular ROS and lipid peroxidase levels were largely abolished (Figures 6A–E), and its regulatory effects on transferrin receptor, GPX4, and xCT were nearly eliminated (Figures 6F–H). Furthermore, the protective effects of LicoA against GSH depletion, MDA production, and Fe^2+^ generation were abrogated (Supplementary Figures S2C–E). Mitochondrial function assays showed that silencing Nrf2 abolished the protective effects of LicoA on cisplatin-induced mitochondrial membrane potential depolarization and structural damage (Figures 6I–M; Supplementary Figures S2F–G). These findings indicate that LicoA suppresses ferroptosis in HK-2 cells in an Nrf2-dependent manner.

### 3.7 LicoA treatment alleviates cisplatin-induced ferroptosis and mitochondrial dysfunction *in vivo*

**FIGURE 7:**
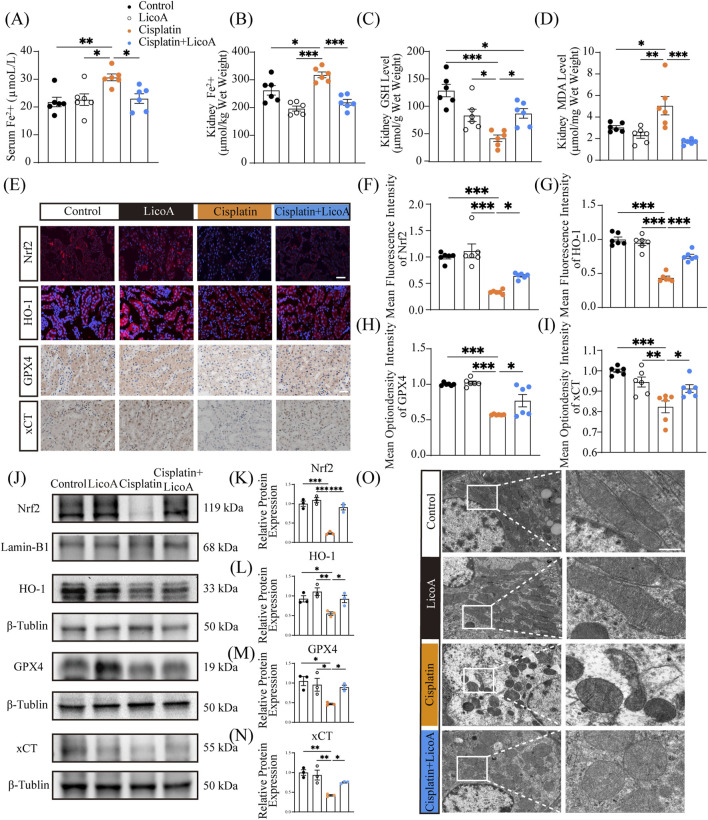
LicoA treatment alleviates cisplatin-induced ferroptosis and mitochondrial dysfunction *in vivo*. **(A,B)** Fe^2+^ levels in serum and kidney. **(C)** GSH levels. **(D)** MDA levels. **(E)** IF staining of Nrf2 and HO-1 in mouse renal and IHC staining of GPX4 and xCT in mouse renal. Scale bar = 50 μm. **(F–I)** The fluorescence intensity of the kidney. **(J–N)** Protein expression levels of Nrf2, HO-1, GPX4, and xCT in renal tissue, n = 3. **(O)** Mitochondrial ultrastructure in renal tissue n = 3. Scale bar = 2 μm. Results are expressed as means ± SEM, all n = 6 per group unless stated.*p < 0.05, **p < 0.01, ***p < 0.001.

Finally, this study validated the effect of LicoA on Nrf2 activation and ferroptosis alleviation using a cisplatin-induced mouse model. LicoA significantly reversed cisplatin-induced GSH depletion, MDA production, and Fe^2+^ accumulation in mouse kidneys (Figures 7B–D). Meanwhile, LicoA also reversed cisplatin-induced Fe^2+^ accumulation in serum (Figure 7A). IF and IHC staining indicated that Nrf2, HO-1, xCT, and GPX4 were significantly upregulated after LicoA treatment (Figures 7D–I), consistent with the results of the Western blot (Figures 7J–N). Besides, TEM observations showed that LicoA significantly mitigated cisplatin-induced mitochondrial damage, including shrinkage, cristae loss, and membrane rupture (Figure 7O). Collectively, LicoA protects against renal ferroptosis by activating the Nrf2/GPX4 antioxidant axis.

## 4 Discussion

Clinical application of cisplatin is often associated with nephrotoxicity (Dasari and Tchounwou, 2014). AKI caused by cisplatin often progresses to CKD due to the periodic chemotherapy regimen. AKI to CKD transition involves complex mechanisms, including tubular epithelial cell injury, oxidative stress, mitochondrial dysfunction, and ferroptosis (Gong et al., 2023; Wang and Zhang, 2022). Despite decades of research, effective clinical interventions to halt cisplatin-induced nephrotoxicity remain scarce (Crona et al., 2017). Among potential therapeutic agents, flavonoids, a class of compounds commonly found in plants, have gained recognition as alternative and adjunctive treatments for various diseases (Serafini et al., 2010; Shen et al., 2022), primarily due to their ability to activate the Nrf2 pathway (Feng et al., 2023; Khan et al., 2020; Lee et al., 2022; Yang et al., 2022). This study established a clinically relevant multi-cycle cisplatin mouse model mimicking AKI progression to CKD, and demonstrated that LicoA, a flavonoid extracted from the root of Ningxia licorice, alleviated cisplatin-induced AKI-to-CKD transition by reducing tubular injury, fibrosis, and renal dysfunction in both *in vivo* and *in vitro* models. Moreover, we selected 5 mg/kg LicoA for the subsequent verification after the preliminary experiments, this dosage selection aligns with findings reported in a previous study (Lv et al., 2024).

Renal tubular epithelial cell (RTEC) injury and death are central to the pathogenesis and progression of CKD (Zhao et al., 2023). In this study, LicoA alleviated cisplatin-induced brush border loss, epithelial shedding, renal tubule dilation, and reduced collagen deposition in tubular basement membranes. Functional assessments revealed cisplatin-induced oliguria without significant proteinuria, indicating that the proximal tubule is the primary site of injury. These results corroborate our previous findings (Wang et al., 2022),and align with those reported in prior studies (Gao et al., 2024; Ozkok and Edelstein, 2014), highlighting the protective effects of licoA on RTECs. Given the high energy demand of RTECs(Han et al., 2016; Li et al., 2021), and their susceptibility to oxidative stress (Schaub et al., 2021), metabolomics analysis was performed. The results demonstrated that cisplatin induced disruption of GSH, which played a critical role in neutralizing free radicals and maintaining cellular redox homeostasis (Diaz-Vivancos et al., 2015; Dickerhof et al., 2017). Notably, LicoA treatment reduced the oxidized form of GSH, while increasing the reduced form, indicating a reduction in cisplatin-induced oxidative stress.

Ferroptosis, a form of iron-dependent cell death characterized by oxidative lipid damage and impaired antioxidant capacity, has emerged as a key player in cisplatin nephrotoxicity (Jiang et al., 2021; Stockwell, 2022; Chen Y. et al., 2024). The dysfunction of system Xc^−^, specifically its subunit xCT, impairs cystine uptake and GSH synthesis, weakening the antioxidant capacity of GPX4, the primary enzyme responsible for reducing lipid hydroperoxides. Ferrous iron (Fe^2+^) drives reactive oxygen species (ROS) generation through Fenton reactions, inducing lipid peroxidation (Inagaki et al., 2016; Xing et al., 2022). Moreover, the vicious cycle of lipid peroxidation and ROS amplification inflicts severe damage to cellular membranes (Rochette et al., 2022; Ursini and Maiorino, 2020). In this study, LicoA treatment reversed the cisplatin-induced downregulation of xCT and GPX4, key players in the antioxidative defense against ferroptosis. Moreover, LicoA effectively inhibited Fe^2+^ accumulation, prevented GSH depletion, reduced malondialdehyde (MDA) and reactive oxygen species (ROS) levels, and suppressed lipid peroxidation in renal tubular epithelial cells. These effects were comparable to those observed with Fer-1, a well-established ferroptosis inhibitor (Scarpellini et al., 2023). This evidence supports that LicoA inhibits cisplatin-induced ferroptosis, highlighting its potential as a therapeutic agent in preventing nephrotoxicity.

Disrupting mitochondrial ROS production effectively prevents ferroptosis (Chen et al., 2021). In addition, mitochondrial dysfunction, marked by diminished mitochondrial size, heightened membrane thickness, absence of cristae in mitochondria, and lower membrane electrical potential, has been identified as a hallmark feature of ferroptosis (Bock and Tait, 2020; Li W. et al., 2022). Our study showed that LicoA co-treatment markedly attenuated these mitochondrial abnormalities, providing evidence linking mitochondrial damage to cisplatin-induced ferroptosis in RTECs.

The central mechanistic insight of this study is that LicoA activates the Nrf2/GPX4 axis, a key pathway in mediating anti-ferroptosis and mitochondrial protection. Molecular docking analysis revealed that LicoA binds effectively to Nrf2, indicating high binding affinity, and treatment with LicoA increased the expression of both Nrf2 and GPX4 in renal cells. The role of Nrf2 in regulating ferroptosis has become increasingly understood, the fully functional Nrf2 maintains cellular iron homeostasis (upregulates ferritin FTH1 and output protein FPN1), ensures antioxidant capacity (activates the System Xc-/GSH/GPX4 axis), and inhibits lipid peroxidation (downregulates ACSL4, etc.) through its transcriptional regulatory network (Gao et al., 2025; Zhou et al., 2024). Interestingly, we found that LicoA increased HO-1 expression. HO-1 has been considered a “double-edged sword” in ferroptosis. Notably, HO-1 upregulation in our setting was accompanied by a decrease in Fe^2+^ levels, suggesting an overall improvement in cellular iron homeostasis. Mechanistically, by activating Nrf2, the combined effects may offset the potential risk of iron overload associated with HO-1 induction, consistent with the findings reported by Luo et al. (Luo et al., 2025). Together, these observations suggest that, LicoA does not simply induce HO-1 in isolation, rather, it triggers a coordinated cellular response that prevents iron accumulation.

The dependence on Nrf2 was unequivocally established through knockdown experiments. Knockdown of Nrf2 in HK-2 cells completely abrogated LicoA’s beneficial effects. It demonstrates that Nrf2 activation is indispensable for LicoA’s renoprotection against cisplatin-induced ferroptosis. The dependence of LicoA’s protective effects on Nrf2 was unequivocally established through Nrf2 knockdown experiments. In HK-2 cells, silencing Nrf2 expression abolished the protective effects of LicoA, confirming that Nrf2 activation is essential for mediating LicoA’s reno protective actions. This finding is consistent with studies using ML385, a pharmacological Nrf2 inhibitor, which reversed the protective effects of various compounds against renal injury and ferroptosis (Zhou et al., 2025; Zhang et al., 2025; Fan et al., 2025) Additionally, RSL3 treatment, which induces ferroptosis through GPX4 downregulation, further validated the involvement of the Nrf2/GPX4 axis in LicoA’s protective mechanism (Liu et al., 2025; Wu et al., 2022).

While our study confirms that LicoA alleviates cisplatin-induced nephrotoxicity by activating the Nrf2/GPX4 axis and inhibiting ferroptosis, there are several limitations that should be addressed in future investigations. First of all, the precise molecular mechanisms by which LicoA regulates Nrf2 remain to be fully elucidated. Structural analysis suggests that LicoA’s α,β-unsaturated carbonyl moiety, characteristic of Michael acceptors, could modify Keap1 cysteine residues, thereby inhibiting Nrf2 ubiquitination and promoting its activation (Nakamura and Miyoshi, 2010). However, further studies are needed to directly confirm this mechanism and explore whether LicoA interacts with other molecular targets that contribute to Nrf2 activation. Besides, the role of GPX4 in mediating the full protective effects of LicoA remains unclear. Future studies using RSL3, a specific inhibitor of GPX4, could provide more definitive evidence on whether LicoA’s renoprotective effects are entirely GPX4-dependent. Additionally, while LicoA demonstrated protective effects comparable to those of Fer-1, we did not investigate potential synergistic effects between LicoA and Fer-1 or other ferroptosis inhibitors. Exploring such combinations could reveal whether multi-targeted approaches, such as combining LicoA with Fer-1 or other ferroptosis inhibitors, offer enhanced therapeutic outcomes in the treatment of cisplatin-induced nephrotoxicity.

**FIGURE 8:**
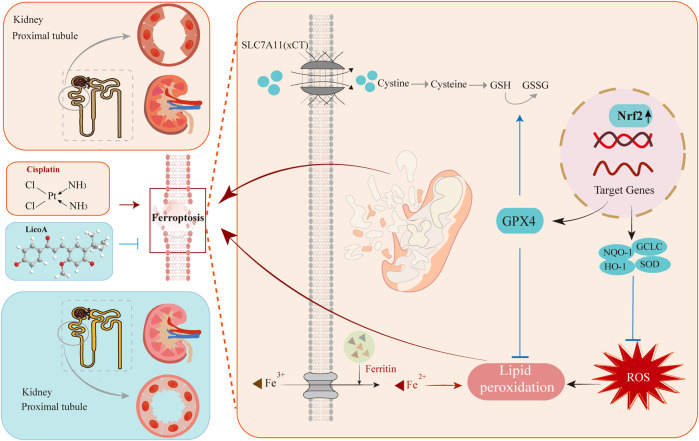
LicoA attenuates cisplatin-induced AKI-to CKD transition by activating Nrf2/GPX4-mediated ferroptosis suppression.

Collectively, this study demonstrates that LicoA alleviates cisplatin-induced renal injury by activating the Nrf2/GPX4 axis. The activation suppresses ferroptosis and ameliorates mitochondrial damage, thereby attenuating the progression from AKI to CKD (Figure 8). These findings reveal novel therapeutic applications for LicoA and position it as a promising candidate for the prevention and treatment of cisplatin-induced nephrotoxicity.

## Data Availability

The data presented in the study are deposited in Figshare, accession number https://doi.org/10.6084/m9.figshare.31940097.
