# Correction: Licochalcone A mitigates cisplatin-induced nephrotoxicity by inhibiting ferroptosis and mitochondrial dysfunction via the Nrf2/GPX4 axis

**DOI:** 10.3389/fphar.2026.1876105

**Published:** 2026-06-12

**Authors:** Mo Han, Feihu Zhang, Mengyuan Li, Qixiang Zhang, Kaidi Shen, Depeng Zhang, Huanke Xu, Fang Zhou, Xinyu Wang

**Affiliations:** 1 Key Laboratory of Drug Metabolism and Pharmacokinetics, State Key Laboratory of Natural Medicines, China Pharmaceutical University, Nanjing, China; 2 Department of Pharmacy and Institute of Clinical Pharmacology, General Hospital of Ningxia Medical University, Yinchuan, China

**Keywords:** cisplatin-induced nephrotoxicity, ferroptosis, LicoA, mitochondrion, Nrf2

There was a mistake in Figure 3 as published. The image for the Cisplatin + Fer-1 group was used incorrectly. The corrected Figure 3 appears below.

**FIGURE 3:**
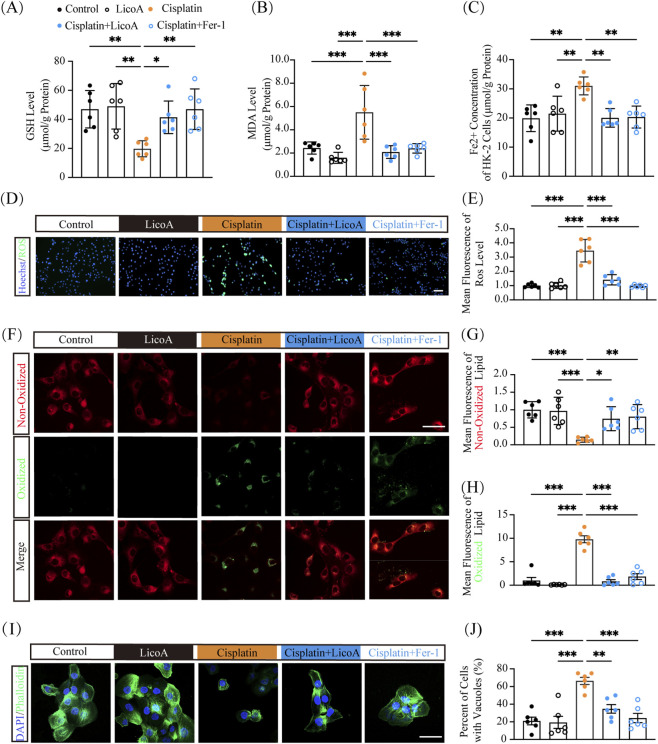
LicoA protects HK-2 cells against cisplatin-induced lipid peroxidation and ferroptosis. **(A)** GSH levels. **(B)** MDA levels. **(C)** Fe^2+^ levels. **(D,E)** ROS levels. **(F–H)** Lipid peroxidation levels. **(I,J)** Co-stained with phalloidin and DAPI. White arrows indicate the bulges of the cell membrane. The number of cells with blistered membranes was calculated. Scale bar = 50 μm. Results are expressed as means ± SEM, all n = 6 per group unless stated. *p < 0.05, **p < 0.01, ***p < 0.001.

There was a mistake in Figure 6 as published. The image for the Cisplatin group under the si-Nrf2 condition were used incorrectly. The corrected Figure 6 appears below.

**FIGURE 6:**
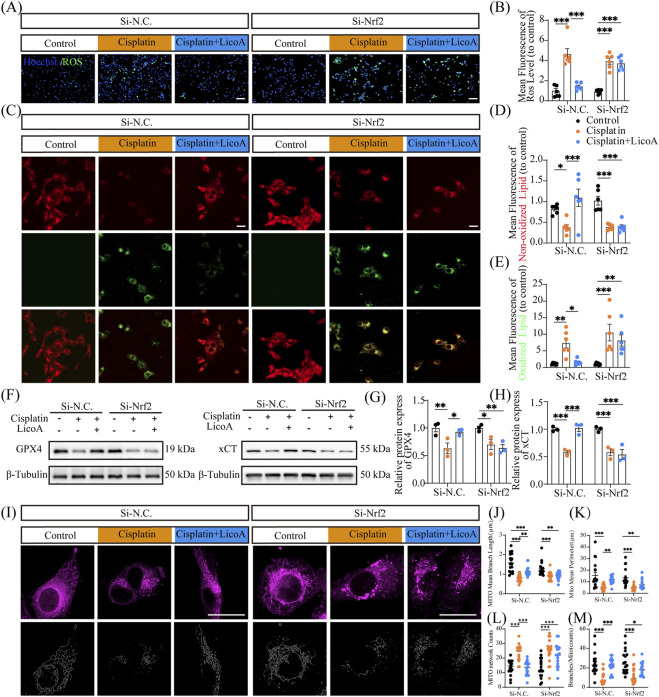
LicoA suppresses ferroptosis in HK-2 cells in an Nrf2-dependent manner. **(A,B)** ROS fluorescence levels. Scale bar = 50 μm, n = 6. **(C–E)** Lipid peroxidation levels. Scale bar = 20 μm, n = 6. **(F–H)** The protein expression of GPX4 and xCT in HK-2 cells, n = 3. **(I)** Mitochondrial morphology, Scale bar = 20 μm. **(J–M)** Mitochondrial mean perimeter, networks, mean branch length, and individual branches. n = 16–25, scale bar = 20 μm. Results are expressed as means ± SEM, *p < 0.05, **p < 0.01, ***p < 0.001.

The original article has been updated.

