# Supplementary material for: Licochalcone A mitigates cisplatin-induced nephrotoxicity by inhibiting ferroptosis and mitochondrial dysfunction via the Nrf2/GPX4 axis
Source: Front Pharmacol. 2026 Apr 17;17:1759824. doi: 10.3389/fphar.2026.1759824 (PMC13132868; doi:10.3389/fphar.2026.1759824)
Supplement: Supplementary file 1 [file Supplementaryfile1.docx]

Supplementary Material


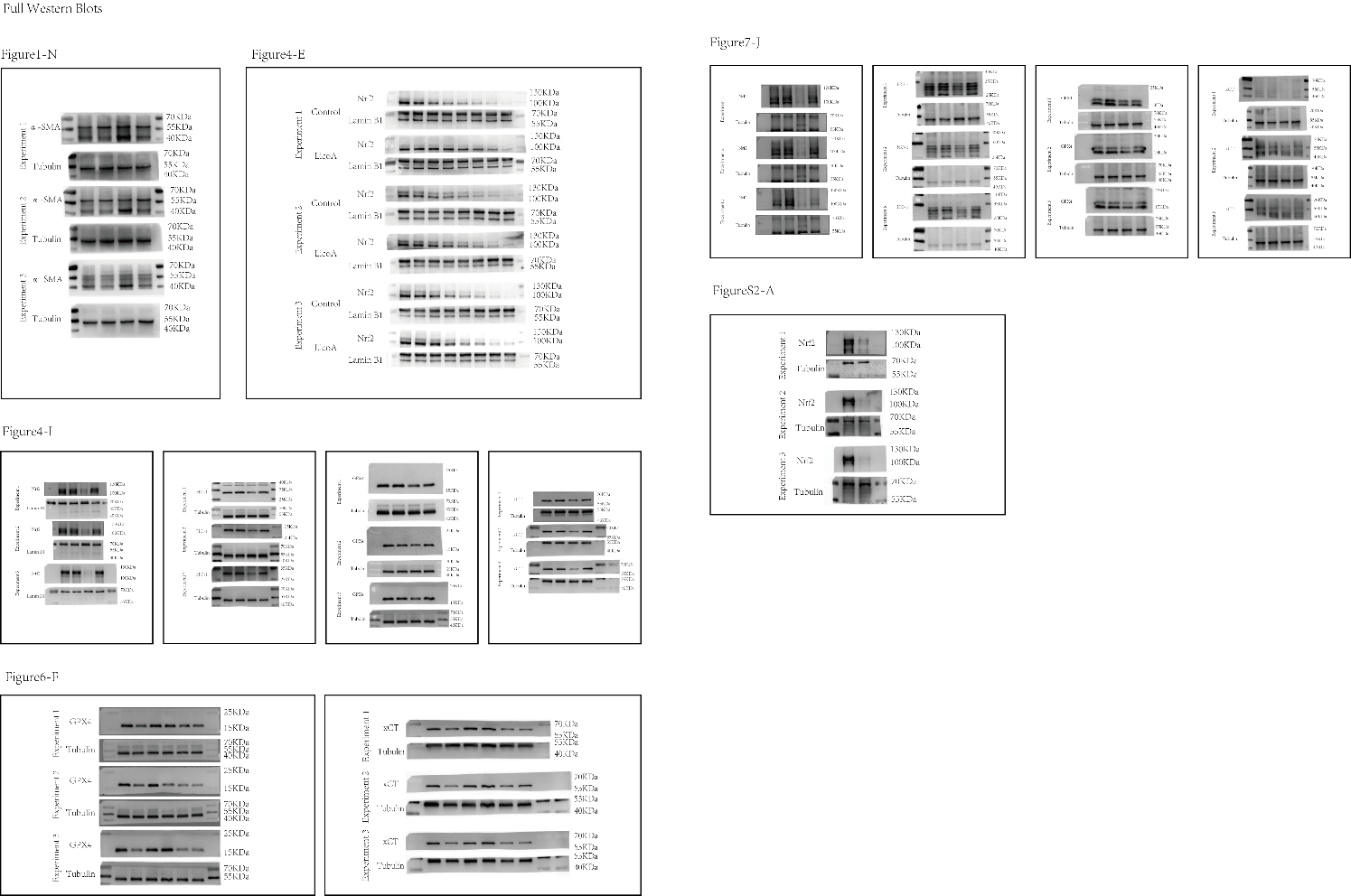
**Supplementary Data**

**Supplementary Figures**


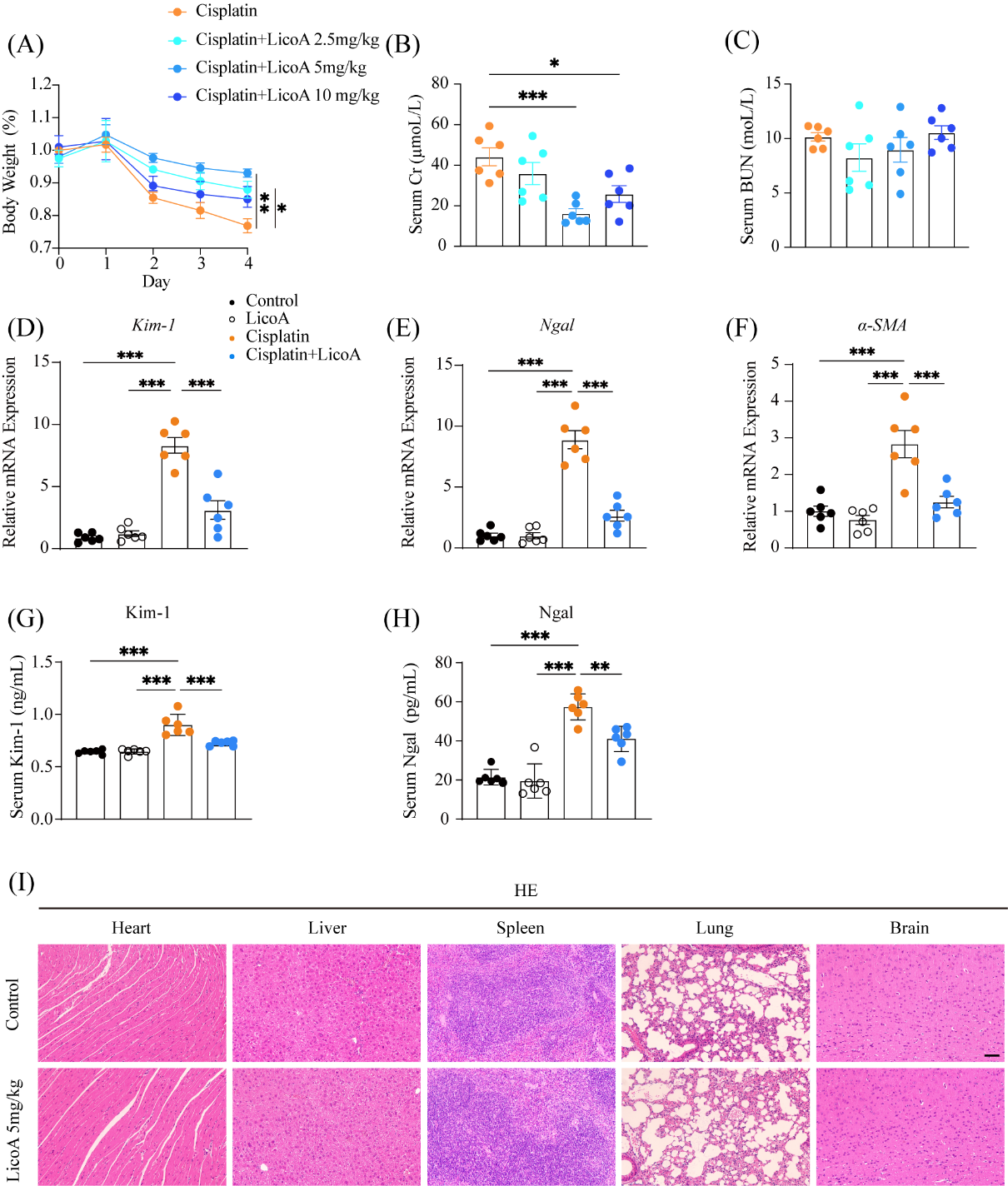


**Fig. S1 The effects of varied doses of LicoA on the body weight and renal function in animal model** (A)Body weight. (B, C) sCr and BUN levels. (D-E) Relative mRNA expression of Kim-1, Ngal and α-SMA.(G,H) Quantitation of Kim-1 and Ngal in serum. (I) The HE staining of tissues from the heart, liver, spleen, lung, and brain of control and 5mg/kg LicoA group mouse. Results are expressed as means ± SEM, all n = 6 per group unless stated. ** p < 0.05, ** p < 0.01, *** p < 0.001.*


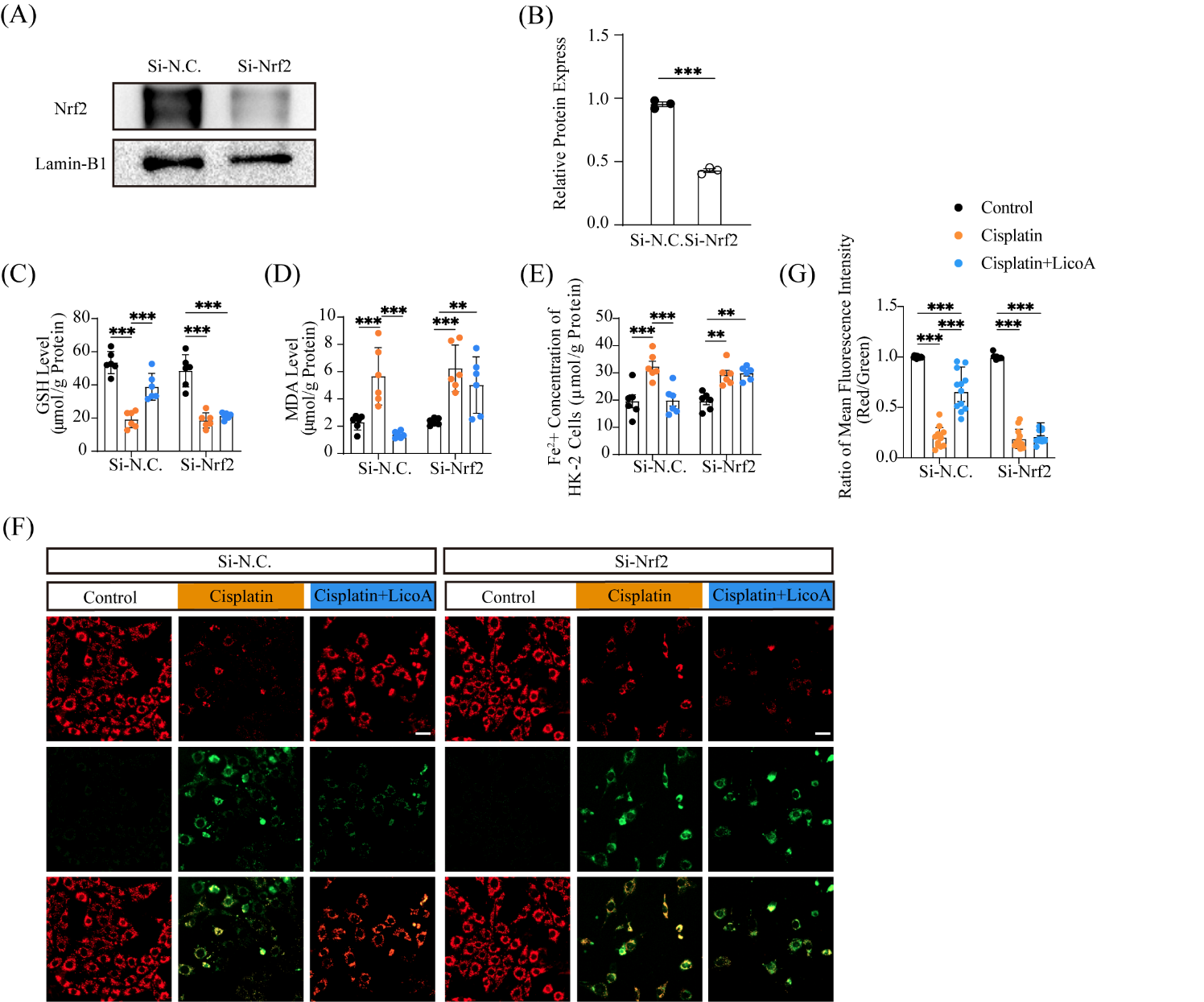


**Fig. S2 The inhibitory effect of LicoA on HK-2 cell ferroptosis is dependent on Nrf2 gene expression**（A-B）Western blotting and statistical data of the Nrf2 protein expression were employed to examine the transfection efficiency of small interfering RNA levels in HK-2 cells, n=3. (C-E) GSH, MDA and Fe2+ levels in HK-2 cells. (F) Assessment of MMP levels in HK-2 cells using JC-1 fluorescent probe. Scale bar = 20 μm. (G) Relative aggregates/monomers fluorescence intensity ratio in HK-2 cells. Results are expressed as means ± SEM, all n = 6 per group unless stated. ** p < 0.05, ** p < 0.01, *** p < 0.001.*
